# Phenotypical T Cell Differentiation Analysis: A Diagnostic and Predictive Tool in the Study of Primary Immunodeficiencies

**DOI:** 10.3389/fimmu.2019.02735

**Published:** 2019-11-29

**Authors:** Enrico Attardi, Silvia Di Cesare, Donato Amodio, Carmela Giancotta, Nicola Cotugno, Cristina Cifaldi, Maria Chiriaco, Paolo Palma, Andrea Finocchi, Gigliola Di Matteo, Paolo Rossi, Caterina Cancrini

**Affiliations:** ^1^Unit of Immunology and Infectious Diseases, Academic Department of Pediatrics, Bambino Gesù Children's Hospital, Rome, Italy; ^2^Department of Experimental and Clinical Medicine, University of Florence, Florence, Italy; ^3^Department of Systems Medicine, University of Rome Tor Vergata, Rome, Italy; ^4^Research Unit of Congenital and Perinatal Infection, Academic Department of Pediatrics, Children's Hospital Bambino Gesù, Rome, Italy

**Keywords:** flow cytometric immunophenotyping, T cell subsets, primary immunodeficiencies, multivariate data analysis, diagnostic markers

## Abstract

Multiparametric flow cytometry (MFC) represents a rapid, highly reproducible, and sensitive diagnostic technology for primary immunodeficiencies (PIDs), which are characterized by a wide range of T cell perturbations and a broad clinical and genetic heterogeneity. MFC data from CD4+ and CD8+ T cell subsets were examined in 100 patients referred for Primary Immunodeficiencies to our center. Naïve, central memory, effector memory, and terminal effector memory cell differentiation stages were defined by the combined expression CD45RA/CD27 for CD4 and CD45RA/CCR7 for CD8. Principal component analysis (PCA), a non-hypothesis driven statistical analysis, was applied to analyze MFC data in order to distinguish the diverse PIDs. Among severe lymphopenic patients, those affected by severe combined and combined immunodeficiency (SCID and CID) segregated in a specific area, reflecting a homogenous, and a more severe T cell impairment, compared to other lymphopenic PID, such as thymectomized and partial DiGeorge syndrome patients. PID patients with predominantly antibody defects were distributed in a heterogeneous pattern, but unexpectedly PCA was able to cluster some patients' resembling CID, hence warning for additional and more extensive diagnostic tests and a diverse clinical management. In conclusion, PCA applied to T cell MFC data might help the physician to estimate the severity of specific PID and to diversify the clinical and diagnostic approach of the patients.

## Introduction

Primary Immunodeficiencies Disorders (PIDs) are a heterogeneous group of congenital disorders, caused by defects in development and/or function of the immune system, associated with an increased susceptibility to infections, immune-dysregulation, and a higher risk of malignancy (1, 2). Currently, about 340 genetic disorders responsible for defects in the immune system have been identified (3). The T cell compartment plays a key role in coordinating innate and adaptive immune responses upon antigen stimulation. Its impairment leads to a broad spectrum of immune diseases, which require rapid and defined diagnosis in order to adopt the targeted therapeutic management. Severe combined immunodeficiency (SCID) are caused by a severe defect in T cells differentiation, variably associated with B cell, natural killer (NK) cell, and/or myeloid lineage impairment, with the first symptoms usually manifesting within the first year of life and the only curative therapy is represented by haematopoietic stem cell transplantation and/or gene therapy for defined diseases (4, 5). Conversely, CID manifest later with a more heterogeneous clinical picture, often associated with immune-dysregulation manifestations (6, 7). Although few observational studies on CID are in progress, a commonly accepted clinical and diagnostic management for these patients has not been defined yet, and it often relies on the local center expertise, rather than on evidence based systematic experiences (8, 9). Moreover, humoral defects, classified as a predominantly antibody defects and common variable immunodeficiency (CVID) are characterized by recurrent infections, hypogammaglobulinemia, poor response to vaccines, and can be associated to diverse T cell abnormalities (10). Currently, the most accepted T cell maturation model suggests a progressive differentiation from naïve T cells to the memory phenotype, ending with the generation of T effector cells (11, 12). Multiparametric flow cytometry (MFC) allows an extensive and detailed characterization of lymphocytes subsets (13, 14). In this study, we analyzed by MFC the T cell immunophenotypes in a large group of PID patients, clinically classified according to ESID (European Society for Immunodeficiencies) criteria (15). T cell subsets frequencies were then investigated by principal component analysis (PCA) in order to test if this analysis could estimate the relative disease severity and could possibly support the clinical and diagnostic approach (16, 17).

## Materials and Methods

### Study Population

Study cohort is composed of 100 patients affected by PID and 30 healthy donors followed at Bambino Gesù Childrens' Hospital between 2013 and 2017 and diagnosed for PID by ESID criteria (15). Patients' data were collected retrospectively and the study groups are described in Table 1 while their clinical and molecular characteristics are reported in Supplementary Tables 1a–c.

**Table 1:** Demographics of the study groups.

**PID groups**	**Number**	**Age (years)** **median—interquartile range**	**Male:Female**
SCID	5	0.8 (0.35–1.6)	5:0
CID	15	12.6 (3–8)	8:7
TE	5	4 (2.5–7)	2:3
DGS	12	12 (9–16)	9:3
LOF STAT3 (AD–HIES)	5	15 (6.7–27.7)	3:2
CGD	13	16 (7–23)	13:0
CVID	16	15.5 (6–18.5)	9:7
Selective IgM Deficiency	1	19	1:0
NEMO Deficiency	2	13 (10–16)	2:0
XLA	5	16 (6–23.5)	5:0
Not determined Agammablobulinemia	1	28	0:1
SIgAD	14	5.5 (3–8.5)	9:5
DOCK8 (AR–HIES) Deficiency	1	2	0:1
SAVI	1	1	0:1
XL–HIGM1	2	12.5 (6–19)	2:0
XIAP Deficiency	1	7	1:0
Undefined T–Defect	1	4	1:0
Healthy Donors	30	6.1 (2.2–12.6)	21:9
Total	130		

*SCID, Severe Combined Immunodeficiency; CID, Combined Immunodeficiency; TE, Thymic Excision; DGS, DiGeorge Syndrome; LOF STAT3 (AD–HIES), Loss Of Function STAT3 (Autosomal Dominat Hyper–IgE Syndrome); CVID, Common Variable Immunodeficiency; CGD, Chronic Granulomatous Disease; XLA, X–linked Agammaglobulinemia; SigAD, Selective IgA deficiency; NEMO Deficiency, Nuclear factor–kappa B Essential Modulator Deficiency; DOCK8 (AR–HIES), Dedicator Of Cytokinesis 8 (Autosomal Recessive Hyper–IgE Syndrome) Deficiency; XL-HIGM1, X-Linked Hyper IgM type 1; SAVI, STING–Associated Vasculopathy with onset in Infancy; XIAP Deficiency, X–Linked Inhibitor of Apoptosis Protein Deficiency*.

Moreover, the study cohort includes 10 patients affected, respectively, by: selective IgM deficiency, NEMO (NF-kappa-B essential modulator) deficiency, not determined agammaglobulinemia, undefined T-defect, DOCK8-deficiency (dedicator of cytokinesis 8 gene), XIAP deficiency (X-linked inhibitor of apoptosis), XL-HIGM1 (X-linked Hyper IgM type 1), STING (STimulator of INterferon Genes) associated vasculopathy with onset in infancy (SAVI). Patients did not receive any corticosteroid treatment or immunosuppressive therapy at enrollment. Patients' median age was 10 years (range 3,6 months−36 years) while healthy donors' median age was 6,1 years (range 5 months−30 years). Healthy donors were immunocompetent individuals. The work was conducted in accordance with the ethical standards of the institutional research committee and with the 1964 Helsinki declaration and its later amendments or comparable ethical standards. Informed consent, approved by the Ethical Committee of the Children's Hospital Bambino Gesù and Policlinico Tor Vergata, was obtained from either patients or their parents/legal guardians, if minors.

### Multiparametric Flow Cytometric Analysis

T cell development can be phenotypically assessed by the combined cell surface expression of CD45RA, CD31, CCR7, and CD27 molecules. CD4 subsets are identified as naïve (CD4+ T_N_: CD45RA+CD27+), central memory (CD4+ T_CM_: CD45RA-CD27+), effector memory (CD4+ T_EM_: CD45RA-CD27–) and terminally differentiated (CD4+ T_EMRA_: CD45RA+CD27-). Moreover, CD4+ naïve T cells coexpressing CD31+ are highly enriched in recent thymic emigrants (RTE), a naïve T CD4+ cell subpopulation that have just egressed the thymus and characterized by higher signal joint T-cell receptor excision circle (sjTREC) content (18, 19). Similarly CD8 subsets are defined by the expression of lymph node homing receptor CCR7 in naïve (CD8+ T_N_: CD45RA+CCR7+), central memory (CD8+ T_CM_: CD45RA-CCR7+), effector memory (CD8+ T_EM_: CD45RA-CCR7-), and terminally differentiated (CD8+ T_EMRA_: CD45RA+CCR7-). All flow cytometric analysis were performed on ethylenediamine tetraacetic acid (EDTA) blood samples within 24 h of venipuncture. After red blood cells lysis with ammonium chloride the lymphocytes were stained with the following previously titrated monoclonal Abs: CD3 PerCP (clone BW264/56, Miltenyi Biotec), CD45RA APC-H7 (clone T6D11, Miltenyi Biotec), CCR7 PE (clone 3D12, Ebioscience), CD4 APC (clone OKT4, Becton Dickinson), CD8 PE-Cy7 (clone RPA-T8, Becton Dickinson), CD19 PE-CY7 (clone SJ25C1, Becton Dickinson), CD16 PE (clone 3G8), CD56 (clone NCAM16.2) PE, CD27 FITC (clone M-T271, Becton Dickinson), TCR alpha-beta APC (clone T10B9, Becton Dickinson), TCR gamma-delta FITC (11F3, Miltenyi Biotec). Cells were incubated with the appropriate antibody cocktail for 30 min at 4°C, washed with PBS and suspended in PBS. At least 50,000 events in the lymphocyte live gate were acquired for each sample. Samples were acquired on FACSCANTO II (BD Biosciences, San Diego, CA, USA) and analyzed with FlowJo software (Tree Star Inc, version 8.8.6, Ashland, Ore). Some patients of our cohort presented with severe lymphopenia and to avoid this bias, we have considered the cell subsets frequencies, instead of absolute counts. Details of the gating strategy are shown in Supplementary Figure 1.

### Statistical Analysis

Unpaired *t*-test was used to compare the patients and controls for variables with normal distribution. For non-parametric variables, the unpaired two-tailed non-parametric Mann-Whitney test was used. All graphical representations and statistical analyses were performed using Prism 6.0 (GraphPad). The relative T subsets frequencies were subjected to PCA analysis, using PAST (PAleontological STatistics, version 3.22, University of Oslo) to visualize and to estimate the correlation among variables.

## Results

**Figure 1:**
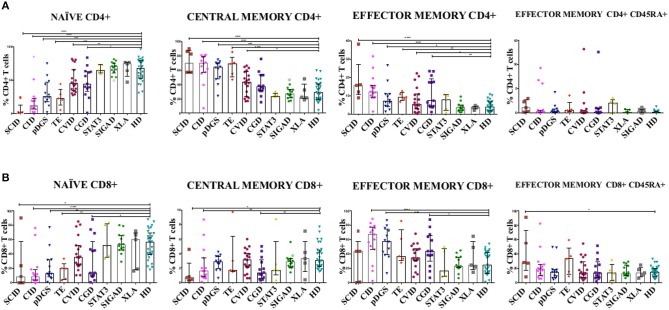
**(A)** CD4+ and **(B)** CD8+ T cell subsets in patients with primary immunodeficiency diseases. Unpaired two-tailed non-parametric Mann-Whitney test was used to compare the patients and controls. The columns and error bars indicate median and interquantile ranges (25°–75°). *P ≤ 0.05, **P ≤ 0.01, ***P ≤ 0.001, ****P ≤ 0.0001. SCID and CID patients showed a severe reduction in frequency of T_N_ CD4+ and CD8+ cells and a corresponding increase in T_CM_ and T_EM_ CD4+ and CD8+, while in CVID patients the same reduction is reported only in CD4+ subsets. DGS and Thymic-excision patients present the same distributions pattern, except for T_CM_ and T_EM_ CD8+. In CGD group there is a reduction in the frequency of T_N_ CD4+ cells and an increase T_EM_ CD4+ cells, while T_N_ and T_CM_ CD8+ T cell compartment are significantly reduced accompanied by T_EM_ CD8+ expansion. Symbol with a cross in the SIgAD groups represents the SIgAD patient with the severe clinical presentation. SCID, Severe Combined Immunodeficiency; CID, Combined Immunodeficiency; TE, Thymic Excision; DGS, DiGeorge Syndrome; STAT3, Loss Of Function STAT3 (Autosomal Dominat Hyper-IgE Syndrome); CVID, Common Variable Immunodeficiency; CGD, Chronic Granulomatous Disease; XLA, X-linked Agammaglobulinemia; SIgAD, Selective IgA deficiency; HD, Healthy Donors.

T subsets frequencies were diversely perturbed in most PIDs in univariate analysis, while those of STAT3, XLA, and SIgAD patients were all comparable to healthy donors. Moreover, no significant differences were evident between all lymphopenic groups (CID, DGS, TE) (Figure 1). T cell subsets frequencies were then interrogated by PCA: STAT3, XLA, and SIgAD groups did not show any evident alteration (Supplementary Figures S4A,B), with the exception of one SIgAD patient with severe autoimmune cytopenia clustering far from the SIgAD group (A13) and two XLA patients (X2 and X4) with T_EM/EMRA_ CD8+ cell expansion (Supplementary Figure 4B).

**Figure 2:**
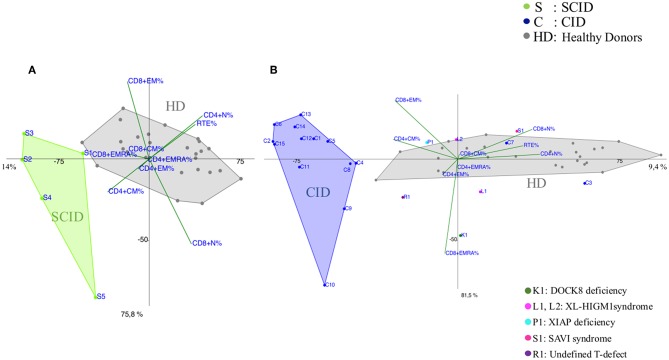
PCA Scatter plots of T cell subpopulations frequencies of **(A)** Severe Combined Immunodeficiency (SCID), **(B)** Combined Immunodeficiency (CID) patients compared to healthy donors (HD). Each sample (subject) is represented by the combination of variables (T subsets) and allocated in a Cartesian space. Samples are plotted, and similarities and differences can be visualized. The overlay of the 2D (2 Dimensional) plot of the scores (subjects) with the 2D plot of the loadings (combination of T subsets) allows the identification of the variables that most contribute to the characterization of a specific subject, since they lie in the same area of the graphs. PCA identifies directions, called principal components, along which the variation in the data is maximal, reported as percentages near the axis. SCID patients are indicated by green dots and the letter S, CID patients by blue dots and the letter C and healthy donors (HD) are represented by gray dots. Individual patients are represented as: C3 and C7 (blue dots) diagnosed as Combined Immunodeficiency patients (CID), K1 (dark green dot) is a DOCK8 Deficiency patient (AR-HIES), L1 and L2 (fuchsia dots) are X-Linked HyperIgM type1 patients (XL-HIGM1), P1 (light blue) is a patient with X-Linked Inhibitor of Apoptosis Protein deficiency (XIAP), S1 (magenta dot) is a SAVI patient (STING-Associated Vasculopathy with onset in Infancy: STING), R1 (purple dot) is a patient with a not determined T cell defect.

Most of the CID patients clearly segregated far from healthy donors, similarly to SCID (Figures 2A,B and Supplementary Figures 2, 5A) and the main discriminating variables were the T_CM_ CD4+ and T_EM_ CD8+ and to a lesser extent T_EMRA_ CD8+ cell subsets, as clearly evident in the CID patients (C9, C13, C14, C15) diagnosed as APDS (activated PI3K delta syndrome) (Figure 2B). While 13 patients out of 15 segregated uniformly, two patients (C3 and C7) classified as CID, with recurrent respiratory infections but in absence of immune dysregulation phenomena, segregated differently: C7 lied inside the HD area and C3 was skewed toward naive cells and low memory subsets in PCA (Figure 2B). Indeed, patient C3 had normal TREC levels, despite a reduction in CD4+CD31+CD45RA+ T cells and an increase in the CD4+CD31-CD27+CD45RA+ (>20%), suggesting a defective T cell maturation. Some other patients were clearly identifiable, like the DOCK8 (AR-HIES) deficiency patient (K1) showing a trend vs. T_EMRA_ CD8+ expansion and the patient (R1) with an undefined T cell defect clustering toward T_EM_ CD4+ differentiation. Conversely, the immunophenotype of the two XL-HIGM1 patients (L1, L2) and XIAP deficiency patient (P1) segregated more closely to HD, although their clinical picture mimicked a CID (Figure 2B). In patient S1, admitted at 14 months of age for a severe dermatitis, chronic diarrhea, and anemia associated with a profound alteration in T cell distribution, PCA showed clearly a peculiar localization near HD area and far from CID group, excluding a combined immunodeficiency. Later targeted next generation sequencing (NGS) analysis revealed a STING (STimulator of INterferon Genes) mutation, justifying her severe course due to a deficiency in the interferon pathway (20).

**Figure 3:**
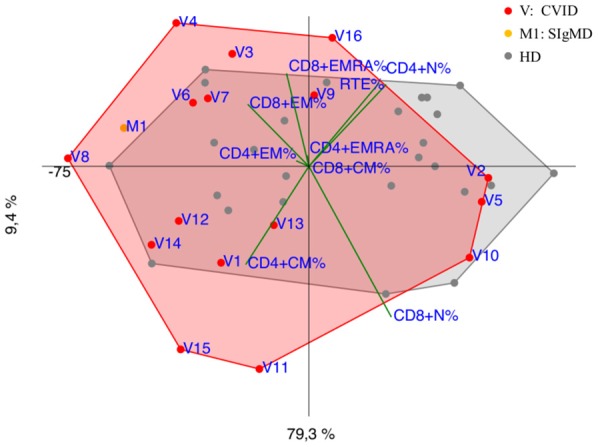
PCA Scatter plot of T cell subpopulations frequencies of CVID patients (shown as red dots and the letter V) and SIgMD patient (orange dot) compared to healthy donors (HD gray dots) show overlapping areas except for V3,V4, V8, V11, V15, V16 and M1.

**Figure 4:**
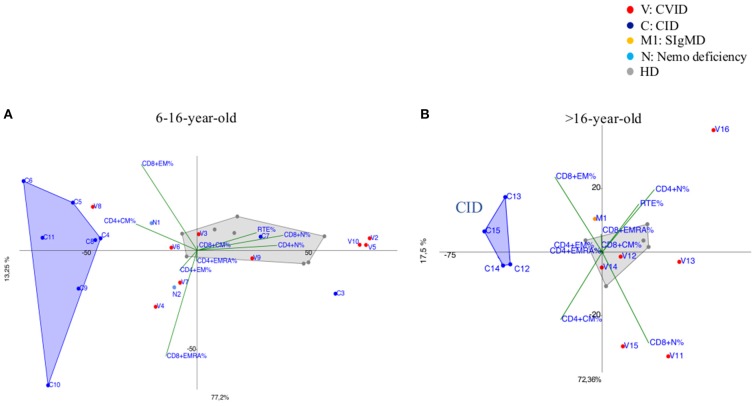
**(A)** 6–16-year-old CVID patients (shown as red dots and with the letter V) compared to 6–16 year old CID patients (distinguished by blue dots and the letter C) and age matched HDs (shown as gray dots). CVID patients (V2, V5, V10) showed immunoprofile with increased T_N_ CD4+ although they do not have the same diagnosis. N1 and N2 (light blue dots) patients bearing a splice site mutation NEMO transcript are differently identified: N1 more vs. T_CM_ CD4+ while N2 showed a trend vs. T_EM/EMRA_ CD8+. **(B)** more than 16-year-old CVID patients (shown as red dots and the letter V) compared to more than 16-year CID patients (drawn as blue dots and the letter C) and age matched HD (shown as gray dots). CID patients (C13, C14, C15) diagnosed as APDS (activated PI3K delta syndrome) and C12 cluster together due to the main discriminating variables such as T_CM_ CD4+ and T_EM_ CD8+ and to T_EMRA_ CD8+ cell subsets. M1 (orange dot) is a selective IgM deficiency (SIgMD) patient with an immunoprofile showing a trend vs. late memory differentiation in both CD4+ and CD8+ subsets.

CVID immunophenotypes did not segregate uniformly (Figure 3), but when analyzed by age groups and compared to CID (Figures 4A,B and Supplementary Figure 3), PCA showed one 4-year-old patient (V1) with a CID-like clinical phenotype, characterized by recurrent infections, bronchiectasis, and facial dysmorfism that clustered far from HD near the CID age matched patients' area (Supplementary Figure 3). Furthermore, two 6–16-year-old CVID patients (V4 and V8, Figure 4A) presented a severe clinical course and could be distinguished by T_EMRA_ CD8+ expansion in V4, probably related to persistent viral infections, and T_CM_ CD4+ and T_EM_ CD8+ in V8; patient V8 developed overtime a MAS (Macrophage Activated Syndrome), leading eventually to death. On the other hand, in the same 6–16 year age range three CVID patients (V2, V5, V10) with increased T_N_ CD4+ clustered nearby but showed diverse molecular diagnosis: in V2 was reported a dominant heterozygous mutation c.2557CNT (p.Arg853^*^) in the *NFKB2* gene, in V5 was detected a mutation in Transmembrane Activator and Calcium-Modulator and Cyclophilin-Ligand Interactor (*TACI*), while V10 is still without a definite diagnosis (Figure 4A).

The immunoprofile of two NEMO deficiency relatives patients in age range 6–16 year (N1 and N2) (Figure 4A) and bearing a splice site mutation in the 59 UTR of the *NEMO* transcript, was skewed differently: N1 more vs. T_CM_ CD4+ while N2 showed a trend vs. T_EM/EMRA_ CD8+.

The older than 16 year CVID patient (V16), mother of patient V2 and bearing the same *NFKB2* mutation of her child, outlied far in the upper right quadrant, influenced by the highly increased T_N_ CD4+ frequencies (Figure 4B). In the same age range two CVID patients (V11, V15), segregated according to their relative T_CM_ CD4+ expansion, maintaining normal T_N_ CD8+ frequencies: V15 carried a Cytotoxic T-Lymphocyte-Associated protein 4 deficiency (CTLA4) while patient V11 is still under investigation (Figure 4B). The single selective IgM deficiency patient (M1) was not characterized by a distinctive differentiation pattern, although segregating outside HD area (Figure 4B).

**Figure 5:**
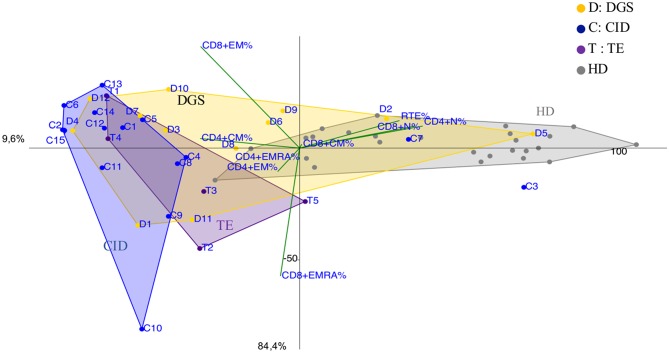
PCA Scatter plots of T cell subpopulations frequencies of DiGeorge Syndrome (DGS, yellow dots and letter D) patients, Thymic Excision patients (TE, distinguished by purple dots and letter T), Combined Immunodeficiency (CID, represented by blue dots and the letter C) patients and healthy donors are shown as gray dots. D1 is the complete DGS patient, D4 and D12 are partial DGS patients with a CID-like clinical presentation.

DGS and Thymic excision patients were distributed in a broad area between controls and CID group (Figure 5 and Supplementary Figure 5B) mirroring the reported variable T cell defect severity and highlighting those with a CID-like phenotype (D4 and D12). Consistently, patient D1, the only one affected by complete DGS, resembling a SCID phenotype, clustered close to SCID area.

**Figure 6:**
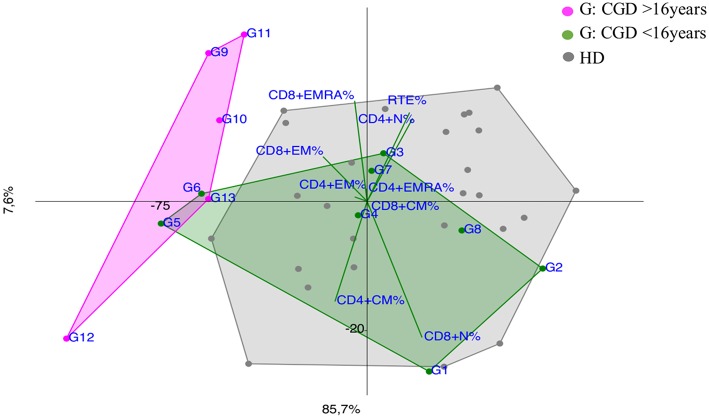
PCA Scatter plots of T cell subpopulations frequencies of Chronic Granulomatous Disease (CGD) patients divided by age: younger CGD patients (G1, G2, G3, G4, G5, G6, G7, G8 represented by green dots) are <16 year old; they cluster inside HD area with the exception of G5 and G6, that are two twin brothers with a severe clinical presentation. The older than 16 year CGD patients (G9, G10, G11, G12, G13 drawn as fuchsia dots), show a CID-like immunoprofile.

Notably, CGD patients showed a trend vs. effector memory subsets, more evident in older patients and in the younger ones (G5 and G6) with a more severe clinical presentation (Figure 6).

## Discussion

Several attempts have been used to categorize PIDs based on clinical manifestations, humoral immune defects and T cell phenotypes. Standardized disease definitions are still lacking in the current classification for PIDs, especially in pediatric age (15, 21). Unsupervised clustering methods applied to immunophenotype data might provide additional information regarding the diagnostic and clinical criteria of PIDs, which do not fulfill any classification. We particularly focused our analysis on specific categories of patients, as well as CID and CVID given their high clinical heterogeneity, which increases the complexity of the diagnostic approach and the clinical management. Indeed, according to PCA analysis, most of CID patients clearly segregated from healthy donors and the principal discriminating variables resulted the T_CM_ CD4^+^ and T_EM_ CD8^+^ and to a lesser extent T_EMRA_ CD8+ cell subsets, suggesting an imbalance between CD4+ helper and CD8+ cytotoxic function in peripheral sites (22, 23). Their expansion in the majority of CID patients is partially explained by a lymphopenia-induced proliferation process (24, 25), but also revealed a trend to an accelerated T cell exhaustion leading to an inefficient immune response and the risk to develop immunedysregulation phenomena (6). This is particularly evident in APDS patients (C9, C13, C14, C15) (Figure 2B), in which their T cell senescence leads to a higher risk of chronic infection, such as EBV replication, and therefore to lymphoproliferative disease/malignancy susceptibility (26). At the same time PCA clearly identified two CID patients (C3, C7) with milder clinical course segregating far from CID area and close to HD (Figure 4A), distinguishing them from those at higher risk to develop severe complications.

A highly heterogeneous pattern of T cell abnormalities has been observed in CVID group (Figures 3, 4A,B and Supplementary Figure 3) and PCA clustered some CVID immunophenotypes in proximity to CID area, providing a clue for a deeper monitoring in these patients. Recent ESID Registry-Working Definitions for Clinical Diagnosis shows that some patients, previously diagnosed as CVID, were reclassified as CID and unclassified antibody deficiency (15, 27). Indeed, V8 patient, initially identified as CVID, showed a marked T_CM_ CD4+ and T_EM_ CD8+ expansion (Figure 4A), suggesting that a more aggressive treatment should have been considered, before developing fatal complications. In patient V15 segregating far from HD due to T_CM_ CD4+ increase (Figure 4B) a *CTLA4* haploinsufficiency responsible for a perturbed T CD4+ cell homeostasis was eventually confirmed by NGS (28).

Interestingly V2 (Figure 4A) and V16 patients (Figure 4B) currently diagnosed as CVID clustered far from either HD and CVID group reflecting their own peculiar *NFKB2* driven differentiation defect (T_N_ cell expansion) (29, 30). NEMO deficiency patients (N1, N2) (Figure 4A) showed a different trend vs. T_CM_ CD4+ and T_EM/EMRA_ CD8+ respectively, confirming the high variability in the clinical and immunological disease expression (31). IgM deficiency patient (M1) (Figure 4B) showed a trend vs. late memory differentiation in both CD4+ and CD8+ subsets that could influence B cell subset, as reported in a larger cohort (32).

As largely described (33), immunoprofiles of patients with partial DGS and Thymic excisions resulted extremely heterogeneuos in PCA (Figure 5), as well as in their clinical course. Patients who underwent to total thymic excision during cardiac surgery in neonatal age show a clinical improvement with age in terms of frequency/severity of infections, suggesting a peripheral recovery of the T-cell compartment (34). Interestingly two patients partial DGS (D4 and D12) with a more severe clinical phenotype (refractory autoimmune cytopenia and recurrent bacterial infections) clustered in CID area suggesting the need of a stricter follow up.

Although CGD is primarily a phagocytes disorder, recent evidence showed a defect in adaptive immunity in both T and B cell compartment (35–37), as shown in PCA by an early T cell senescence evident in older CGD patients (Figure 6), probably related to chronic inflammation. PCA could be useful to consider and to monitor an immunomodulating treatment whenever necessary in CGD patients to reduce the T cell exhaustion (38).

No evident alterations and specific segregation were detected in XL-HIGM1, XIAP, LOF STAT3 (AD-HIES), and SIgAD patients (Supplementary Figures 4A,B), except for one SIgAD patient (A13) with a severe autoimmune cytopenia which segregated outside the HD area (39–42). Although reduction in CD4+ memory T cell subsets was previously reported in XLA patients (43), this data was not confirmed in our limited cohort and a longer follow up is necessary. Only two XLA patients (X2 and X4) segregated accordingly to their T_EM/EMRA_ CD8+ cell expansion, likely associated to recent infections (Supplementary Figure 4B).

In order to test the PCA potentiality, we applied it in three patients (K1, R1, and S1) with a severe clinical presentation not easily classifiable (Figure 2B and Table 1). Patient K1, with a history of endocarditis, vasculitis and sepsis, apparently normal T cell count, showed a high and evident terminal effector CD8+ T cells expansion and later DOCK8 deletion was detected using multiple genetic approaches (44, 45). Patient R1, with a history of interstitial pneumonia and lymphocytes lung infiltration, showed T_EM_ CD4+ expansion, alerting us for a severe but still undefined T cell defect. Finally, child S1 with a picture mimicking a CID, in PCA segregated close to healthy donors' area, ruling out a severe immunodeficiency. Mutation in STING gene was detected by NGS targeted panel for autoinflammatory inborn error allowing the start of a specific treatment (46).

## Conclusions

The multivariate data processing techniques could be used as a diagnostic and prognostic tool to identify peculiar immune profiles, to screen atypical PID with higher risk for severe disease progression and monitor the response to personalized therapeutic approaches.

## Data Availability Statement

The datasets generated for this study are available on request to the corresponding author.

## Author Contributions

SD performed the experiments, analyzed, and interpreted the results and wrote the manuscript. EA analyzed and interpreted the results and wrote the manuscript. GD and CCi performed the molecular analysis. EA, NC, DA, CG, CCi, PP, and AF provided or referred clinical samples and patient's clinical data. EA, SD, and CCa designed the work, participated to the study design and data interpretation. NC, DA, MC, GD, CCi, and PR revised critically the manuscript.

### Conflict of Interest

The authors declare that the research was conducted in the absence of any commercial or financial relationships that could be construed as a potential conflict of interest.

## Supplementary Material

Supplementary Figure 1 Naiv¨e and memory T-cell subsets gating strategy. PBMC from lysed whole blood were gated on live lymphocytes and identified as CD45+. Then after sequential gating on CD3+CD4+ subset, the naiv¨e (T_N_) cells were distinguished as CD45RA+CD27+, central memory cells (T_CM_) as CD45RA-CD27+, effector memory cells (T_EM_) as CD45RA-CD27-, and terminally differentiated cells (T_EMRA_) as CD45RA+CD27-. Among CD3+CD4+ T cells, recent thymic emigrants (RTE) were identified as CD31++CD45RA+. Similarly after gating on CD3+CD8+ subset, the naiv¨e (T_N_) cells were gated as CD45RA+CCR7+, central memory cells (T_CM_) as CD45RA-CCR7+, effector memory cells (T_EM_) as CD45RA-CCR7-, and terminally differentiated cells (T_EMRA_) as CD45RA+CCR7-.

Supplementary Figure 2 PCA Scatter plot of T cell subpopulations frequencies in SCID patients and HD <6-year-old. Healthy donors <6-year-old are represented by gray dots and SCID patients by green dots and the letter S. PCA identifies directions along which the variation in the data is maximal, in this analysis represented by T_CM_ CD4+ cells inversely correlated to T_N_ CD4+ as variables that most contribute to the characterization of different subjects.

Supplementary Figure 3 Less than 6-year-old Common Variable Immunodeficiency (CVID) aged patient (V1) indicated by red dot and the letter V compared to aged matched Combined Immunodeficiency (CID) patients, represented by blue dots and the letter C and age matched healthy donors (HD) shown as gray dots.

Supplementary Figure 4 PCA Scatter plot of T cell subpopulations frequencies in **(A)** Loss of Function STAT3 (LOF STAT3) (Autosomal Dominant Hyper-IgE Syndrome) (AD-HIES) patients represented by green dots and the letter E and healthy donors by gray dots, showing the complete overlapping of the areas. **(B)** X-linked Agammaglobulinemia (XLA) patients by light blue dots and the letter X, Selective IgA deficiency (SIgAD) patients by yellow dots and the letter A, one not defined Agammaglobulinemia patient (n.d) by brown dot and letter Y, healthy donors are represented by gray dots. The X2 and X4 patients' immunoprofiles segregated outside HD area, accordingly to their T_EM/EMRA_ CD8+ cell expansion. Not defined Agammaglobulinemia (Y1) patient clustered in the HD area, not revealing any peculiar pattern.

Supplementary Figure 5 PCA Scatter plots of T cell subpopulations frequencies of **(A)** Severe Combined Immunodeficiency (SCID) compared to Combined Immunodeficiency (CID) patients. SCID patients are indicated by green dots and the letter S and CID patients by blue dots and the letter C. **(B)** DiGeorge Syndrome (DGS) patients (represented by yellow dots and letter D), Thymic excision patients (TE) (by purple dots and letter T) compared to CID patients (by blue dots and the letter C).

Supplementary Tables 1a–c Clinical and molecular diagnosis of the patients.

## Abbreviations

SCID: severe combined immunodeficiency
CID: combined immunodeficiency
TE: thymic excision
DGS: DiGeorge syndrome
LOF STAT3 (AD-HIES): loss of function STAT3 (autosomal dominant hyper-IgE syndrome)
CVID: common variable immunodeficiency
CGD: chronic granulomatous disease
XLA: X-linked agammaglobulinemia
SIgAD: selective IgA deficiency
NEMO deficiency: nuclear factor-kappa B essential modulator deficiency
DOCK8 (AR-HIES) deficiency: dedicator Of cytokinesis 8 (autosomal recessive hyper-IgE syndrome) deficiency
XL-HIGM1: X-linked HyperIgM type1
SAVI: STING-associated vasculopathy with onset in infancy
XIAP: X-linked inhibitor of apoptosis protein
TACI: transmembrane activator and calcium-modulator and cyclophilin-ligand lnteractor
CTLA4: cytotoxic T-lymphocyte-associated protein 4.
