# Supplementary figures and images for: Phenotypical T Cell Differentiation Analysis: A Diagnostic and Predictive Tool in the Study of Primary Immunodeficiencies
Source: Front Immunol. 2019 Nov 29;10:2735. doi: 10.3389/fimmu.2019.02735 (PMC6896983; doi:10.3389/fimmu.2019.02735)

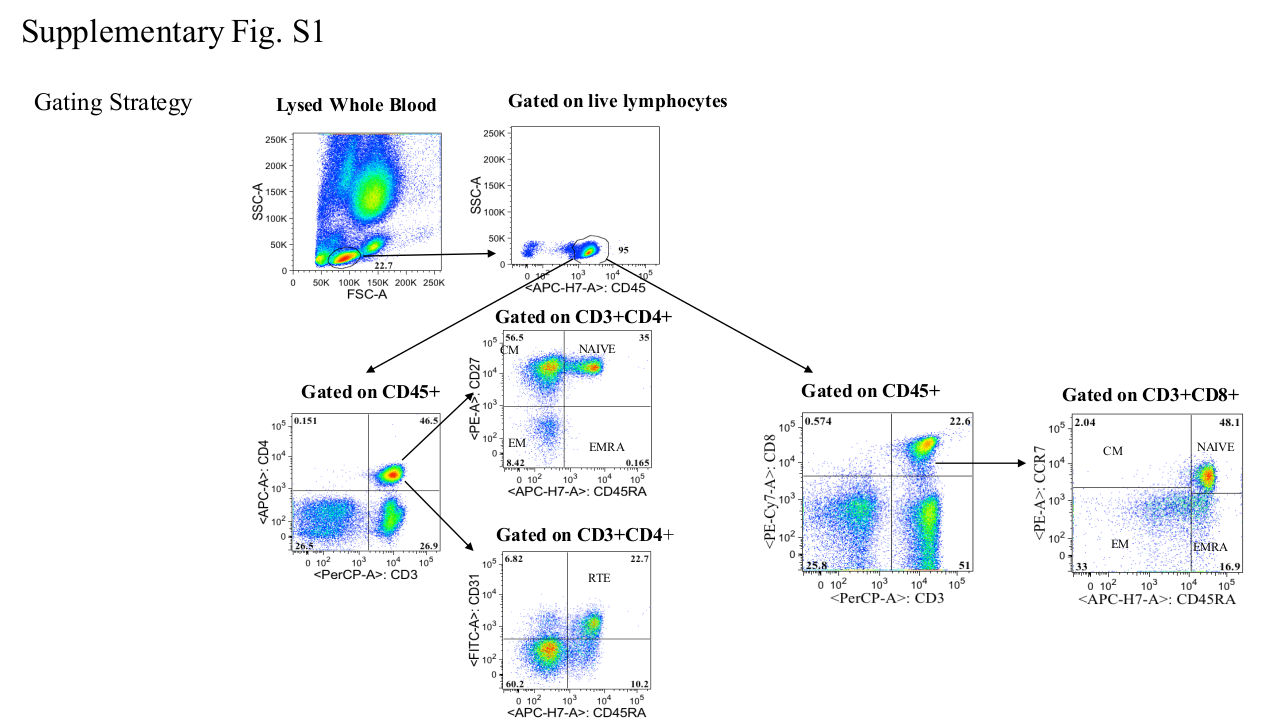

Supplement: Supplementary Figure 1 — Naiv¨e and memory T-cell subsets gating strategy. PBMC from lysed whole blood were gated on live lymphocytes and identified as CD45+. Then after sequential gating on CD3+CD4+ subset, the naiv¨e (TN) cells were distinguished as CD45RA+CD27+, central memory cells (TCM) as CD45RA-CD27+, effector memory cells (TEM) as CD45RA-CD27-, and terminally differentiated cells (TEMRA) as CD45RA+CD27-. Among CD3+CD4+ T cells, recent thymic emigrants (RTE) were identified as CD31++CD45RA+. Similarly after gating on CD3+CD8+ subset, the naiv¨e (TN) cells were gated as CD45RA+CCR7+, central memory cells (TCM) as CD45RA-CCR7+, effector memory cells (TEM) as CD45RA-CCR7-, and terminally differentiated cells (TEMRA) as CD45RA+CCR7-. [file Image_1.TIF]

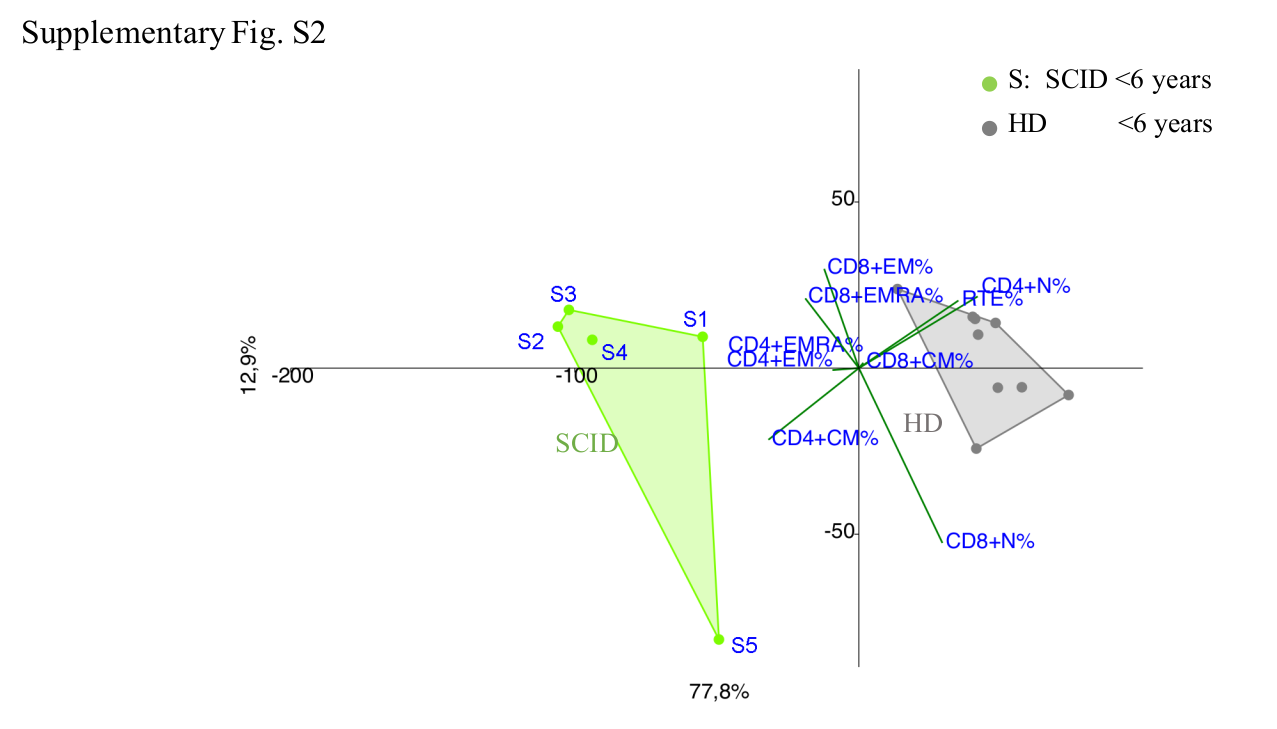

Supplement: Supplementary Figure 2 — PCA Scatter plot of T cell subpopulations frequencies in SCID patients and HD <6-year-old. Healthy donors <6-year-old are represented by gray dots and SCID patients by green dots and the letter S. PCA identifies directions along which the variation in the data is maximal, in this analysis represented by TCM CD4+ cells inversely correlated to TN CD4+ as variables that most contribute to the characterization of different subjects. [file Image_2.TIF]

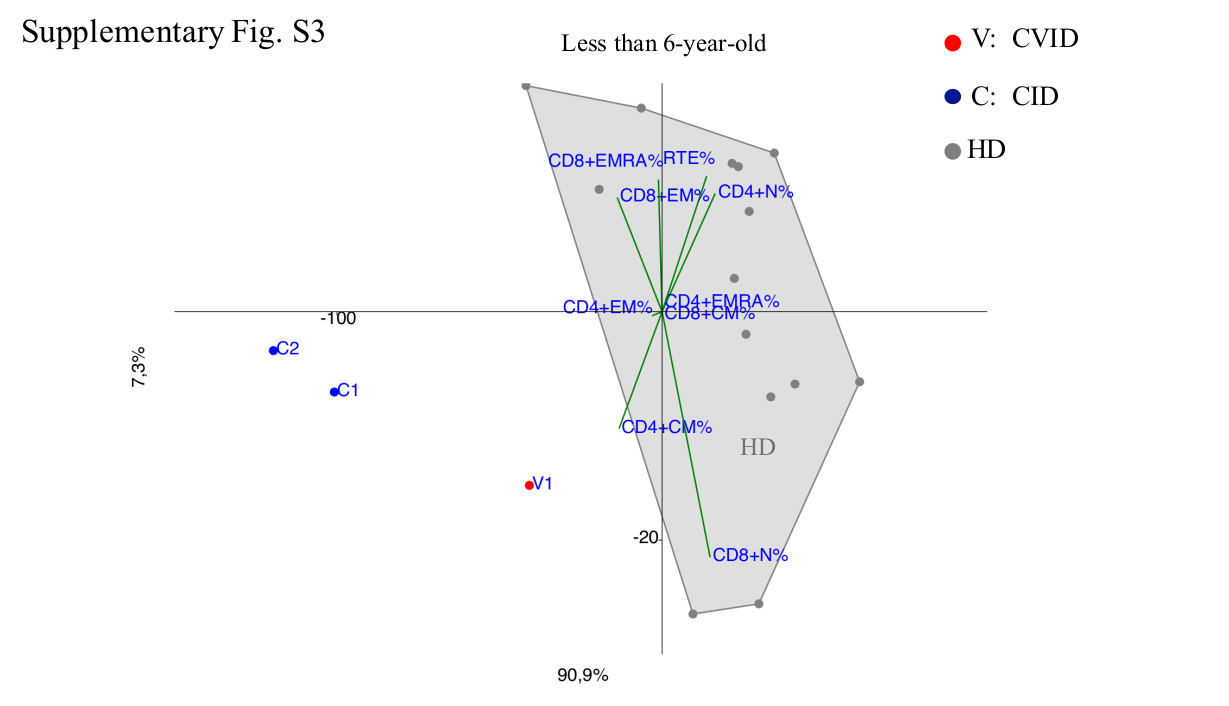

Supplement: Supplementary Figure 3 — Less than 6-year-old Common Variable Immunodeficiency (CVID) aged patient (V1) indicated by red dot and the letter V compared to aged matched Combined Immunodeficiency (CID) patients, represented by blue dots and the letter C and age matched healthy donors (HD) shown as gray dots. [file Image_3.TIF]

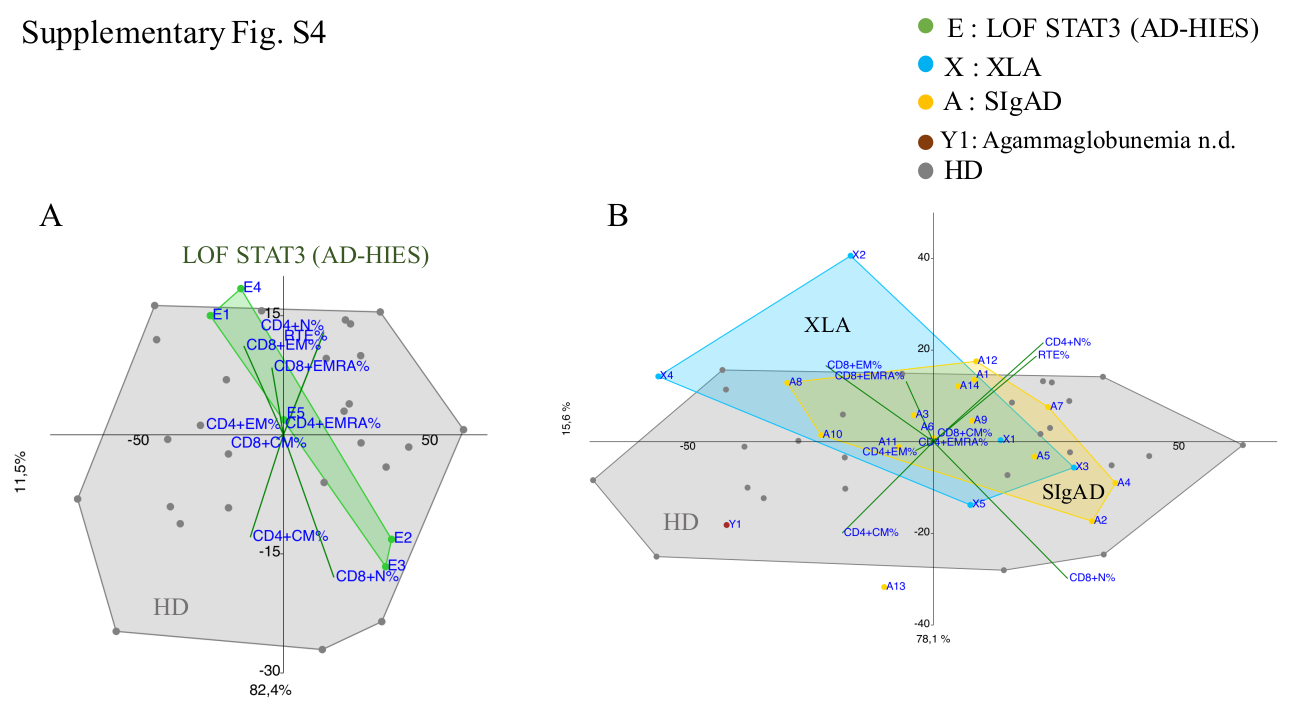

Supplement: Supplementary Figure 4 — PCA Scatter plot of T cell subpopulations frequencies in (A) Loss of Function STAT3 (LOF STAT3) (Autosomal Dominant Hyper-IgE Syndrome) (AD-HIES) patients represented by green dots and the letter E and healthy donors by gray dots, showing the complete overlapping of the areas. (B) X-linked Agammaglobulinemia (XLA) patients by light blue dots and the letter X, Selective IgA deficiency (SIgAD) patients by yellow dots and the letter A, one not defined Agammaglobulinemia patient (n.d) by brown dot and letter Y, healthy donors are represented by gray dots. The X2 and X4 patients' immunoprofiles segregated outside HD area, accordingly to their TEM/EMRA CD8+ cell expansion. Not defined Agammaglobulinemia (Y1) patient clustered in the HD area, not revealing any peculiar pattern. [file Image_4.TIF]

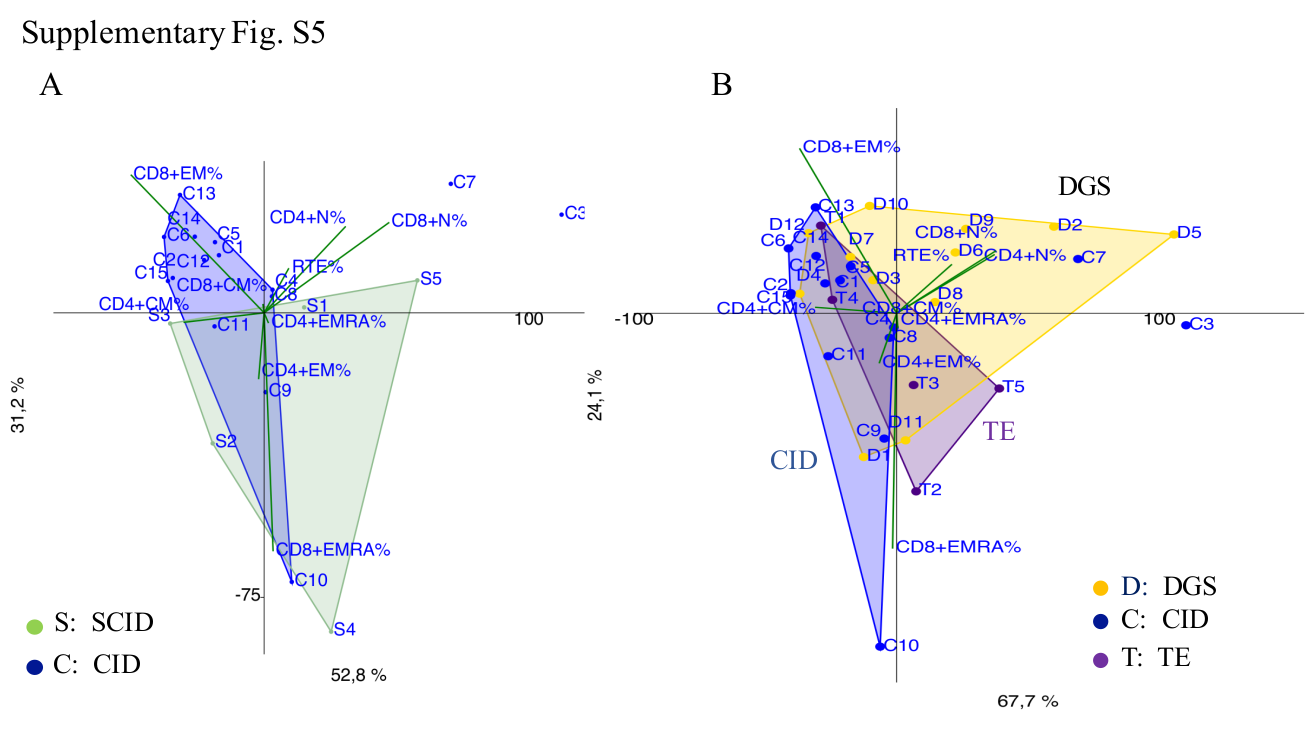

Supplement: Supplementary Figure 5 — PCA Scatter plots of T cell subpopulations frequencies of (A) Severe Combined Immunodeficiency (SCID) compared to Combined Immunodeficiency (CID) patients. SCID patients are indicated by green dots and the letter S and CID patients by blue dots and the letter C. (B) DiGeorge Syndrome (DGS) patients (represented by yellow dots and letter D), Thymic excision patients (TE) (by purple dots and letter T) compared to CID patients (by blue dots and the letter C). [file Image_5.TIF]

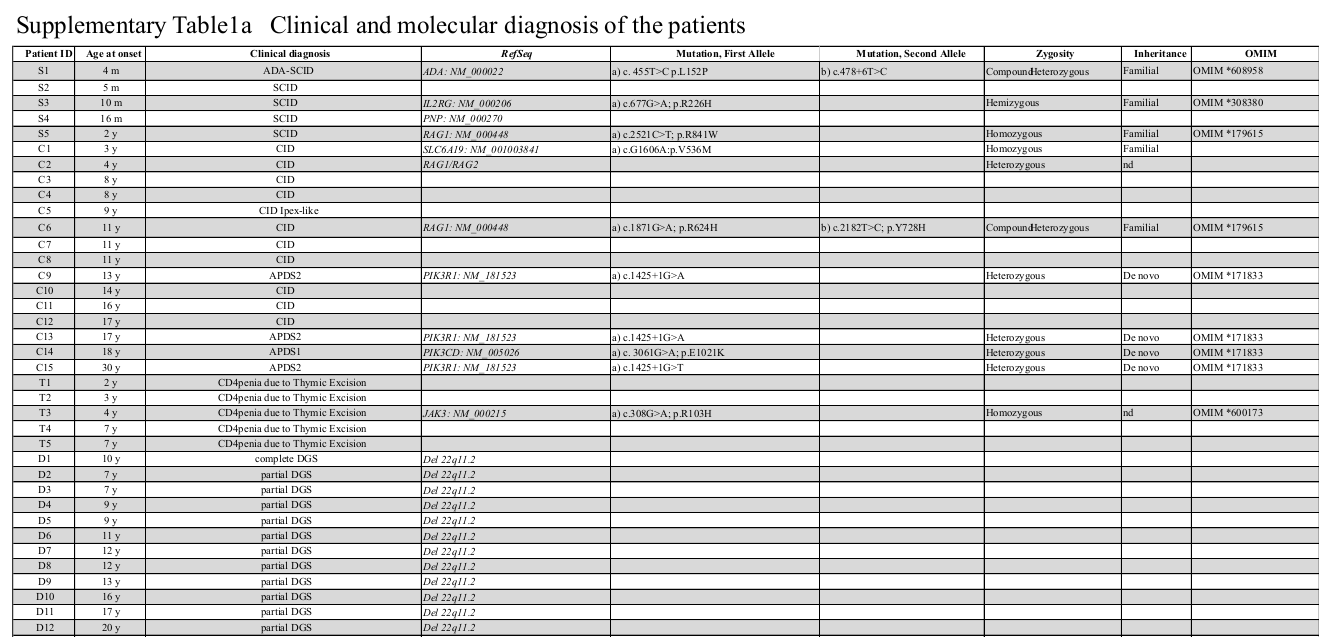

Supplement: Supplementary Tables 1a–c — Clinical and molecular diagnosis of the patients. [file Image_6.TIF]

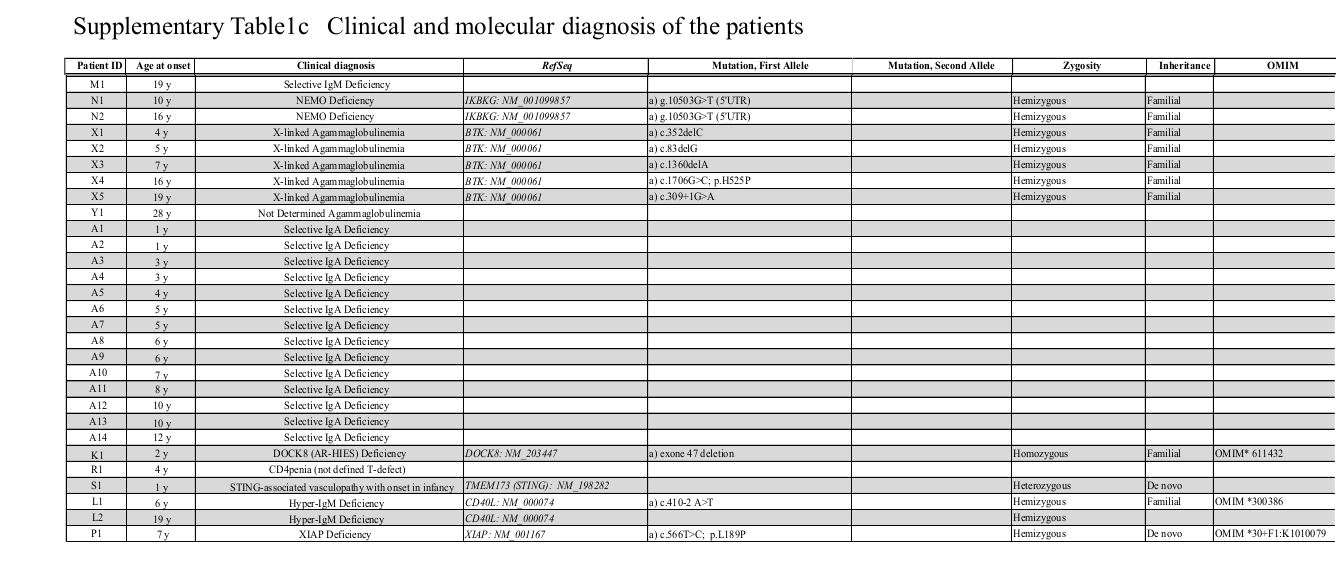

Supplement: Supplementary file 7 [file Image_7.TIF]

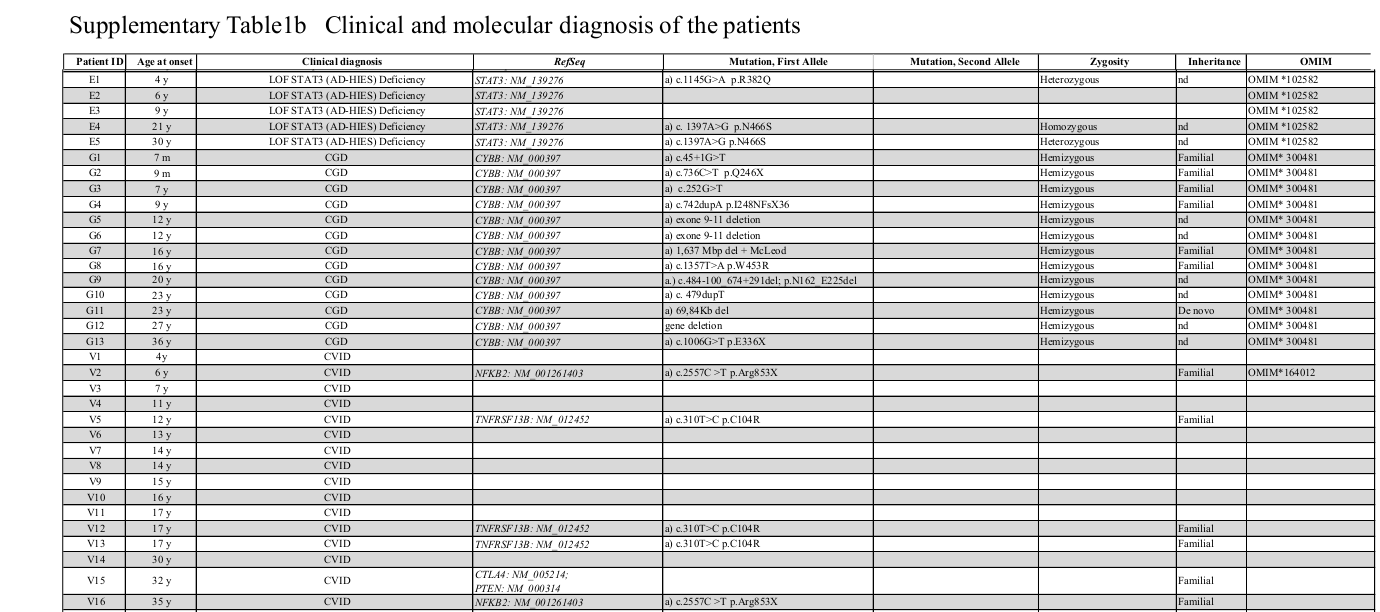

Supplement: Supplementary file 8 [file Image_8.TIF]
